# Appraising the quality of tools used to record patient-reported outcomes in users of augmentative and alternative communication (AAC): a systematic review

**DOI:** 10.1007/s11136-019-02228-3

**Published:** 2019-06-18

**Authors:** Katherine Broomfield, Deborah Harrop, Simon Judge, Georgina Jones, Karen Sage

**Affiliations:** 1 Gloucestershire Care Services NHS Trust, Gloucester, UK; 2 Sheffield Hallam University, Sheffield, UK; 3 Barnsley Hospital NHS Foundation Trust, Barnsley, UK; 4 Leeds Beckett University, Leeds, UK; 5 Bristol Speech and Language Therapy Research Unit, Pines and Steps, Southmead Hospital, Westbury on Trym, Bristol, BS10 5NB UK

**Keywords:** Augmentative and alternative communication, AAC, Communication aids, Patient-reported outcome measures, PROMs, Systematic review

## Abstract

**Purpose:**

People who have complex communication needs (CCN), and who use augmentative and alternative communication (AAC) to help them express themselves, can be difficult to engage in decision making about their healthcare. The purpose of this review was to identify what patient-reported outcome measures (PROMs) have been employed with people who use AAC. Of the tools identified, the review aimed to establish what conceptual frameworks were used and how the reports describe completion of the PROM.

**Methods:**

A systematic literature review was carried out. A pre-defined set of search terms was entered into five main health and education databases. Titles and abstracts were sifted for relevance. Full text papers were screened against inclusion/exclusion criteria. Data pertaining to the type and nature of the PROM used was extracted. Complementary data sources were analysed to construct a narrative synthesis of the papers identified.

**Results:**

Within 15 papers that met the review criteria, 25 PROMs were used with people who rely on AAC comprising of 15 separate measures. The conceptual frameworks for 12 of these tools were reported from which 62 items, or concepts being measured, were identified. Following synthesis of these items, 9 conceptual domains and 11 sub-domains were generated. Limited information was available about who completed the PROM nor how much, if any, support they received.

**Conclusions:**

No PROM that has been developed specifically for people who use AAC was identified by this review. Of the tools that have been used with people who use AAC, the concepts measured were broad and varied. The quality of reporting concerning who completed the PROM was limited, undermining the trustworthiness of many of the studies.

## Introduction

Augmentative and alternative communication (AAC) refers to strategies used to support people who have complex communication needs characterised by difficulties with speech and/or language production in spoken or written modes [1]. They range from simple, paper-based systems consisting of single pages or books of words, phrases or pictures (known as ‘low-tech AAC’) to more complex electronic or computer-based systems (known as ‘high-tech AAC’). High-tech AAC can be used to produce synthesised speech of messages stored within them or entered into them by the person who uses AAC or their family, carer or AAC professional [1].

The population who use AAC is diverse and consists of people with multiple and complex physical and cognitive difficulties [2]. People who use AAC may have communication difficulties from birth associated with conditions such as cerebral palsy or acquire difficulties as an adult following a stroke, head injury or from a degenerative condition such as Parkinson’s [1]. Approximately one in 150 people in England (0.5% of the population) could benefit from using AAC [3].

Children and young people use AAC to support them to access education and enable them to build peer relationships [4]. Adults and older people use AAC to maintain relationships, occupation and to avoid social isolation [5]. However, AAC is a type of assistive technology (AT) and obsolescence and non-use of AT has been identified as a concern for a long time [6]. There is evidence to suggest that AAC is also at risk of being under-utilised or abandoned if, for example, people have limited access to support or training, devices are not maintained, or if there is poor fit between the AAC device and the individual using it [7]. Some researchers have identified a connection between the level of engagement of the AT end-user and the overall use of AT solutions [8, 9]. Understanding that an individual’s needs and priorities are at the heart of clinical assessment, can foster improved engagement in healthcare [10] and can lead to improved health outcomes [11].

Patient-reported outcome measures (PROMs) refer to tools that have been designed to provide information on the status of a patient’s health condition [12]. A PROM should measure a specific concept (or set of concepts), known as a conceptual framework, which has been developed with relevance to an intended population [12]. The use of PROMs is not considered credible unless there is evidence that it has been validated with the population of interest [12]. The purpose of PROMs is to get patients’ own assessment of their health or their health-related quality of life (typically concepts relating to emotional health and physical functioning) and therefore patients usually complete these directly [13]. PROMs are usually in the form of questionnaires and are typically used as evaluation tools. They can also be of value as part of the clinical interview and assessment [14]. For example, the information can be used by clinicians as a mechanism for engaging patients in decision-making about their healthcare at assessment but also during goal-setting, treatment planning and evaluation. Completion of a PROM might also contribute to helping patients to feel cared for, providing a framework for structuring patients’ discussions with their clinician [15].

People who have communication difficulties are inherently difficult to engage in traditional mechanisms for collaborative decision-making [16]. Difficulties in understanding spoken or written words as well as physical limitations often co-occur in conditions associated with the speech impairments necessitating AAC. These additional difficulties may make the completion of paper questionnaires or engaging in interviews challenging. People who require AAC may also have additional cognitive limitations or learning difficulties which require adapted materials and information methods [17]. Communication interactions may need to be navigated via multi-modal approaches, involving visual and pictorial support, facilitated by experienced and skilled communication partners. The nature of how information is attained (i.e. authorship) is critical to understanding the extent to which the person who uses AAC has truly been involved in providing it.

Improving collaboration, engagement and person-centredness in AAC service provision has the potential to improve AAC use and reduce the risk that use of the assistive technology is discontinued by better matching technology to the needs and expectations of the individual [18]. Yet people who have communication difficulties who may benefit from AAC and their families are rarely involved in decision-making relating to AAC [19]. There are no consistently used patient-reported outcome measures specifically for AAC [20]. Neither is there any consensus about what constitutes a successful outcome from AAC from the perspective of the person who uses on it [21]. The lack of appropriate support for, or engagement with, people who use AAC can cause frustration, disillusionment and finally abandonment of equipment [22]. Effective use of suitable PROMs by professionals working with people who use AAC has the potential to enable inclusivity by capturing important outcomes, providing targeted training and support, and evaluating success from the perspective of the people who use AAC.

This systematic review aims to identify:

1. What tools have been used to collect patient-reported outcomes in people who use AAC?
2. What are the conceptual frameworks, domains of interest and validity of the available tools?
3. What methods are employed to enable authorship (i.e. completion) of PROMs by people who use AAC?

## Methods

A systematic review protocol to address the review question was developed and registered on PROSPERO. A list of search terms related to (a) AAC/AT, (b) PROM and (c) communication disorders was generated based on search terms used in reviews on similar populations [21]. The search strategy was deliberately broad initially, including terms relating to communication and AT, to ensure that all measures were captured (for a full copy of the search strategy, see PROSPERO: https://www.crd.york.ac.uk/prospero/display_record.php?RecordID=80567).

### Searches

Databases searches were carried out using CINAHL (EBSCO), ERIC (ProQuest), MEDLINE (EBSCO), PsycINFO (ProQuest), and Scopus (Elsevier) from inception to January 2018.

### Inclusion/exclusion

Studies were included based on meeting the following six criteria: (i) people who had communication difficulties; (ii) people aged 12 years old and above (and where data for this population can be disambiguated); (iii) people who had used an external aid to facilitate communication; (iv) the tools identified had been used to record outcomes from the perspective of the person using the aid (including but not exclusively: published scales and measures, questionnaires, software, descriptive outcomes, and author developed tools), (v) all study types (i.e., qualitative, quantitative, and mixed methods studies); and finally (vi) all contexts outside of acute and fixed-term rehabilitation hospitals. Papers not written in English, where English translations were not available were excluded for pragmatic reasons. Papers reporting participants as having severe intellectual disability [23] and participants who have significant cognitive impairment affecting reasoning and judgement were excluded as it was judged that they would be unable to complete a PROM. Participants with autistic spectrum disorders (ASD) or social communication difficulties were not included as clinicians within the review team decided that outcomes from AAC may be significantly different within this sub-population. Papers concerning participants who exclusively use gesture (including sign language), facial expression, or postures were not included as they did not include the use of an external aid to facilitate communication. Papers concerning assistive devices not commonly issued by AAC services were excluded such as: brain–computer interface; speech recognition technology; assistive devices for hearing or visual impairment. Papers reporting reviews, editorials, and opinion paper were not included as they were not reporting on primary data.

### Screening

**Fig. 1:**
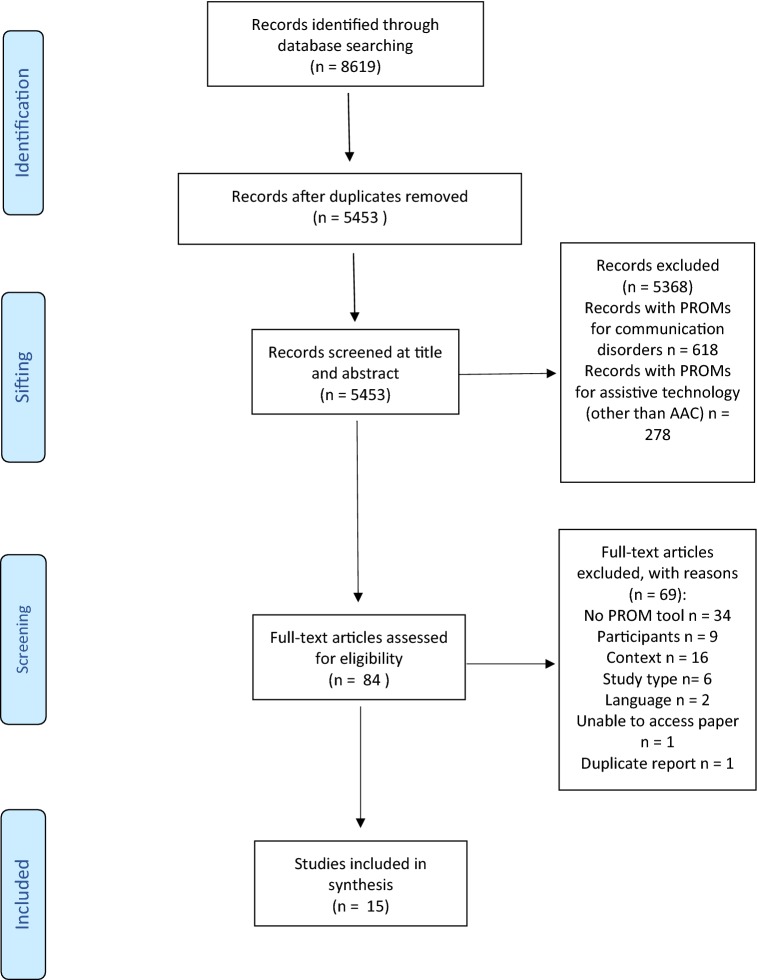
PRISMA 2009 flow diagram

Titles and abstracts of all papers were screened for relevance by one author (KB), and 10% of the papers were independently checked by a second author (DH). There was a 5% inclusion/exclusion disagreement between the screeners which was resolved by discussion. Full-text papers were screened by the first author and 10% of these were checked by a second author (DH) with no disagreement. The PRISMA flow chart (Fig. 1) summarises the screening process.

### Quality appraisal

Papers included in the review were appraised for quality using the Mixed Methods Appraisal Tool (MMAT) [24]. This quality appraisal tool was selected due to the range of study types included in this review. The MMAT enables reviewers to assess the quality of a paper on a 5-point scale from 0 to 4, depending on what information is available in the report. Quality appraisal was carried out by one author (KB) and checked for consistency by a second author (KS). These two authors compared and discussed independent scoring of the papers until consensus was agreed.

### Data extraction

Data were extracted from the included papers and documented in a table designed for the purpose of this review. The table was piloted by two authors (KB, KS) to ensure that it captured the necessary information and to ensure a consistent approach was being used. Data were gathered pertaining to the study details, methods, participant demographics and characteristics, intervention, outcomes, the PROM used (including domains, conceptual framework and content validity as reported in the paper), and author reported strengths and limitations of the tools and study.

### Data synthesis

A complementary approach to data analysis was adopted for this mixed-methods review [25]. A range of data pertaining to population characteristics, study type and PROM used, was extracted (Table 1) as well as descriptive data about PROMs; validity, authorship (Table 2) and concepts measured (Table 3). These data enabled analysis on the depth and breadth of information available as opposed to specifically the type or source of the data [25]. The use of complementary data sources (i.e. about the study and the PROM) enabled the authors to construct a narrative synthesis [26] of the range of PROMs used with people who use AAC. Once the PROMs had been identified and descriptive data about those PROMs had been extracted, the specific concepts (items measured by the PROMs) were tabulated (Table 3). One author (KB) reviewed the concepts and carried out a preliminary synthesis by grouping them into over-arching domains. These domains were then presented to a second author (KS), the strengths and limitations of the groupings were discussed, and a secondary synthesis was agreed and is presented as a final set of domains (see Table 4).

**Table 1:** Study characteristics

Study title	Authors	Year	Journal/publication	Country	Study design	Relevant sample	Ages of participants	Aetiology	Quality of paper (MMAT)	Patient-reported Outcome Measure (PROM)
Quality of life in Amyotrophic Lateral Sclerosis patients and caregivers: Impact of assistive communication from early stages.	Ana Londral, Anabela Pinto, Susana Pinto, Luis Azevedo, Mamede De Carvalho	2015	Muscle and Nerve	Portugal	Quantitative: Longitudinal cohort study	27	39 to 83	ALS (Amyotrophic Lateral Sclerosis)	3	McGill QoL (MQoL), Communication Effectiveness Index - modified (CETI-m)
Communication aids for the speech impaired: Cost and quality of life outcomes assessment programs provided by specialist communication aids centres in the UK	Keith Tolley, Brenda Leese, Ken Wright, Sue Hennessy, Corrine Rowley, Janet Stowe, Ann Chamberlain	1995	International Journal of Technology Assessment in Healthcare	UK	Quantitative: case series	78	1 to 65+	CP (Cerebral Palsy, ALS, Stroke, Other	0	Author developed tool
Electronic mail: Service from afar for individuals with physical disabilities	Elizabeth MacKinnon, Gillian King, Tamzin Cathers and John Scott	1995	Augmentative and Alternative Communication	USA	Quantitative: RCT pilot	16	7 to 25	CP, other	1	Author developed tool
Eye tracking communication devices in amyotrophic lateral sclerosis: Impact on disability and quality of life	Marco Caligari, Macro Godi, Simone Guglielmetti, Franco Franchignoni & Antonio Nardone	2013	Amyotrophic Lateral Sclerosis and Frontotemporal degeneration	Italy	Quantitative: descriptive	35	43 to 60	ALS	2	Individually-prioritised Problem Assessment (IPPA); Quebec User Evaluation of Satisfaction (QUEST); Psycho-social Impact of Assistive Devices (PIADs)
Living with locked-in syndrome: an explorative study on health care situation, communication and quality of life.	L. Snoeys, G. Vanhoof and E. Manders	2013	Disability and Rehabilitation	Belgium	Mixed methods: descriptive	8	16 to 57	LIS (Locked in Syndrome)	1	SF-36 (adapted)
Long-term outcomes for individuals who use augmentative and alternative communication: Part I – what is a “good” outcome?	Shelley K. Lund & Janice Light	2009	Augmentative and Alternative Communication	USA	Mixed methods; sequential exploratory	7	19 to 23	CP	1	Quality of Life Profile - Physical Disabilities (QoLP-PD); International Classification of Functioning (ICF) levels of functioning
Home trials of a speech synthesiser in severe dysarthria: patterns of use, satisfaction and utility of word prediction	Isabelle Laffont; Claude Dumas; Delphine Pozzi; Maria Ruquet;Anne Calire Tissier; Frederic Lofaso; Olivier Dixien	2007	Journal of Rehabilitation Medicine	France	Quantitative: case series	10	9 to 66	CP, ALS, LIS, brain injury	3	QUEST
Speech therapy and communication device: Impact on quality of life and mood in patients with amyotrophic lateral sclerosis	Soja Korner, Michael Siniawski, Katja Kollewe, Klaus Jan Rath, Klaus Krampfl, Antonia Zapf, Reinhard Dengler and Susanne Petri	2012	Amyotrophic Lateral Sclerosis and Frontotemporal degeneration	Germany	Quantitative: cohort	38	Mean age 63.7	ALS	0	SF36
Investigating the success factors of expert users to inform device development	Simon Judge, Zoe Clarke, Mark Hawley	2011	Everyday Technology for Independence and Care. G.J.Gelderblom et al. (Eds)	UK	Qualitative: description	0	0	0	0	CETI-m
An eye-tracking assistive device improves the quality of life for ALS patients and reduces the care giver burden	Chi-Shin Hwang, Ho-Hsiu Weng, Li-Fen Wang, Chon-Haw Tsai & Hao-Teng Chang	2014	Journal of Motor Behaviour	Taiwan	Quantitative: Case control	20	38-85	ALS	0	Taiwanese Depression Questionnaire (TDQ); Amyotrophic Lateral Sclerosis-Quality of Life -Revised (ALS-QoL-R)
Post-school quality of life for individuals with developmental disabilities who use AAC	Bruce Hamm and Pat Mirenda	2006	Augmentative and Alternative Communication	Canada	Mixed methods: embedded design	4	19-24	CP,	2	QoLP-PD; communication survey
Talking Mats in a discussion group for people with Huntington’s Disease	Lisa Hallberg, Elin Mallgren, Lena Hartelius & Ulrika Ferm	2013	Disability and Rehabilitation: Assistive technology	Sweden	Mixed methods; sequential explanatory	4	23-67	HD (Huntington’s disease)	2	Author developed tool
When are high-tech communicators effective in Parkinson’s disease?	Giorgio Ferriero, Marco Caligari, Gianpaolo Ronconi and Franco Franchignoni	2012	International Journal of Rehabilitation Research	Italy	Quantitative: Case study	1	63	PD (Parkinson’s)	0	QUEST; PIADS
Exploring communication assistants as an option for increasing communication access to communities for people who use augmentative communication	Barbara Collier, Donna McGhie-Richmond & Hazel Self	2010	Augmentative and Alternative Communication	Canada	Qualitative: exploratory	9	26-70	CP	1	Author developed tool
Assessment of computer aided assistive technology: analysis of outcomes and costs	Ursula Hass, Agneta Andersson, Hakan Brodin and Jan Persson	1997	Augmentative and Alternative Communication	Sweden	Quantitative: cohort	74	5 to 74	Not specified	3	Rosser classification (modified); Nottingham Health Profile (NHP); Measure of Goal fulfilment

**Table 2:** PROMs and significant descriptive data

PROM/tool used	Number of reports using tool	Aetiology/ies^1^	Type of AAC (H = computer of electronic based devices, L = paper-based systems)	Aggregated sample size	Content validity, as reported in the study	Number of papers reporting authorship (who completed the PROM):			
						Patient	Researcher	Collaborative	Not reported
McGill Quality of Life Questionnaire (MQoL)	1	ALS	Tablet with text 2 speech software (H)	27	Yes: for people with ALS				1
Communication Effectiveness Index - Modified (CETI-m)	2	ALS	Tablet with text 2 speech software (H)	27	Yes: original CETI valid for people with aphasia		1		1
Author developed tool^a^	4	CP, MND, HD, Other	Range of hi-tech and low-tech, Talking Mats (H, L)	107	No		3		1
Individually Prioritised Problem Assessment (IPPA)	1	ALS	Eye-tracking, E-Tran (H, L)	35	Not reported				1
Psychosocial Impact of Assistive Devices (PIADs)	2	ALS, PD,	Eye-tracking, E-Tran, Computer-based, direct access (H, L)	36	Not reported	1			1
Quebec User Evaluation of Satisfaction (QUEST)	3	PD, CP, ALS, LIS, brain damage	Eye-tracking, E-Tran, Computer-based, direct access (H, L)	60	Not reported	1			2
SF-36 (adapted)	2	LIS, ALS	Alphabet boards, text 2 speech, computers, direct and indirect access (H, L)	46	Yes	2			
QoL profile for people with sensory and physical disabilities (QoLP-PD)	2	CP	Communication boards, switch access AAC, direct access AAC, eye-gaze (H, L)	11	Yes	1		1	1
International Classification of Functioning levels of impairment	1	CP	Communication boards, switch access AAC, direct access AAC (H, L)	7	Not reported				1
Arc’s self-determination scale	1	CP	Communication boards, switch access AAC, direct access AAC (H, L)	7	Not reported				1
Revised ALS Specific Quality of Life Instrument (ALS-QoL-r)	1	ALS	Spring track (eye-gaze), phoneme board (H, L)	20	Not reported		1		
Taiwan Depression Scale (TDQ)	1	ALS	Spring track (eye-gaze), phoneme board (H, L)	20	Not reported		1		
Communication questionnaire	1	CP	Communication book, eye gaze (H, L)	4	Yes			1	
Rosser classification (modified),	1	Not specified	Computer-based (H)	74	Not reported			1	
Nottingham Health Profile (NHP)	1	Not specified	Computer-based (H)	74	Yes			1	
Measure of goal fulfilment	1	Not specified	Computer-based (H)	74	Not reported			1	

*ALS* amyotrophic lateral sclerosis, *CP* cerebral palsy, *MND* motor neurone disease, *HD* huntington’s disease, *PD* Parkinson’s, *LIS* locked in syndrome

^a^Author developed tools are those that were designed specifically for the study by the authors and have not been published separately

**Table 3:** Items/concepts measured by PROMs (as reported in the review papers)

PROM	Content items									
IPPA	Basic needs	Sharing new information	Social closeness							
QUEST	Satisfaction with device									
PIADs	Functional independence	QoL	Well-being	Competence	Adaptability	Self-esteem				
SF36	Physical functioning	Physical role	Bodily pain	General health	Vitality	Social functioning	Emotional role	Mental health		
QoLP-PD	Mental well being	Physical well-being	Social involvement	Community involvement	Access to resources	Life enhancing opportunities	Physical	Psychological	Spiritual	Social
Communication survey	Communication with people	Communication in specific places	Communication functions							
ICF levels of impairment	Receptive language	Reading comprehension	Communication interaction skills	Linguistic competency	Functional communication	Education and vocational achievement	Self-determination	Quality of life	Contextual factors	
NHP	Sleep	Energy	Social isolation	Pain						
Rosser (modified)	Personal care	Usual activities	Communication	Pain	Satisfaction	Dependence				
Goal fulfilment	Handling equipment	Functional ability (reading and writing)	Activities or roles							
TDQ	Physical	Sentimental	Cognitive							
ALS-QoL-r	Negative emotion	Interaction with people and the environment	Religiosity	Intimacy	Physical symptoms & bulbar function

**Table 4:** Synthesis of PROM domains

Patient reported outcome measure		Domains																			
		Communication		Cognitive	Role		Health				QoL	Physical				Social		Spiritual	AAC equipment		
	Sub-domain:	Language Skills	Function		Activities	Actualisation	General	Mental	Emotional well-being	Sleep		Energy	Symptoms	Pain	Independence	Functioning	Relationships		Competence	Adaptability	Satisfaction
IPPA			x												x		x				
QUEST																					x
PIADs							x		x		x				x				x	x	
SF36					x		x	x	x			x	x		x	x					
QoLP-PD				x		x	x	x							x	x	x	x			
Communication Survey			x													x	x				
ICF levels of functioning		x	x			x					x					x	x				
NHP										x		x		x			x				
Rosser Classification (modified)			x		x									x	x						x
Measure of Goal fulfilment		x			x														x		
TDQ				x					x				x								
ALS-QoL-r									x				x			x	x	x			

## Results

A total of 5453 titles were identified by the search strategy after duplicates were removed. Of these 5453, 84 full text journals were read and screened (see PRISMA flow in Fig. 1 for details). The search resulted in 15 papers that met the inclusion/exclusion criteria for this review. These 15 papers were quality appraised using the MMAT tool. Of the 15 papers: five reports scored 0, four reports scored 1, three reports scored 2, and three reports scored 3. See Table 1 for a list of study characteristics and the results of the MMAT appraisal process.

### Tools

Twenty-five instances of PROM use with people who use AAC were reported across these 15 studies, comprising 15 different published tools. Author-developed tools were used in four studies [27–30]. A summary of the characteristics of the PROMs identified by the review can be found in Table 2.

Ten of the 15 PROMs were used with participants who relied on either or both low-tech and high-tech communication aids. The McGill Quality of Life Questionnaire (MQoL), Communication Effectiveness Index—modified (CETI-m) [31, 32], Rosser classification (modified), Nottingham Health Profile (NHP) and the measure of goal fulfilment [33] were only used with people who rely exclusively on high-tech communication.

### Content

Of the 15 PROMs identified, reports documented the conceptual frameworks for 12 and consequently, 62 items were identified that the tools measured. For example, the SF-36 consists of items pertaining to eight health concepts: physical functioning, bodily pain, role limitations due to physical health problems, role limitations due to personal or emotional problems, emotional well-being, social functioning, energy/fatigue, and general health perceptions [34]. Following synthesis of all the items measured by each tool, nine conceptual domains were generated: communication, cognition, role, health, quality of life, physical, social, spirituality, and AAC equipment. Within each of these domains, several sub-domains were created which represent areas of nuance or distinction within the main domains. For example, within ‘health’, there are four sub-domains: general, mental, emotional well-being, and sleep. In total, 20 individual domains or sub-domains were synthesised.

### Validity

Authors were rarely explicit about their rationale for selecting particular PROMs. For articles that reported on their rationale, PROMs were used to capture different aspects of AAC technical function and the purposes for which AAC was used. Some papers were evaluating a specific intervention [28, 29, 35], whereas others were reporting longer-term outcomes for AAC [21, 27, 36].

The content validity, as reported in the review papers, was extracted but there were no reported instances of tools that have been psychometrically evaluated specifically in relation to people who use AAC. Some authors did however report on their rationale for amending certain PROMs. Londral el al. [31] reported that the MQoL has been used in various studies with people who have Amyotrophic Lateral Sclerosis (ALS) and that the CETI, a measure for evaluating functional communication in people with aphasia, has been demonstrated to be sensitive to changes over time (in a population of people who have aphasia). However, a modified version of the CETI was used in this particular study (to account for its use with a different population) but there was no information about whether the modification was also sensitive to change. The SF-36 has been used widely in health research and with a range of different conditions but was, again, modified in the study that reported on the validity of the tool [34], with no comment about the nature nor the validity of the modification. The Quality of Life Profile- Physical Disabilities (QOLP-PD) and the communication questionnaire were both used in the study by Hamm and Mirenda [36] and the report cites a previous study which had evaluated efficacy of both tools with a similar population, but the sample size of this study was small (nine) so validity cannot be ascertained.

### Authorship

Authorship (i.e. who completed the PROM) was extracted, capturing how data gathering was adapted to accommodate any physical, cognitive or communication difficulties. There were five examples where the PROMs used were reportedly completed by the participant directly (Quebec User Evaluation of Satisfaction (QUEST) and Psychosocial Impact of Assistive Devices (PIADs) [37]; SF-36 [36, 38]; QOLP-PD and International Classification of Functioning (ICP) levels [21]); six examples where tools were completed by the researcher (author developed tools [28–30]; CETI-M [32]; ALS-Qol-r and Taiwanese Depression Questionnaire (TDQ) [39]) and five examples of tools being completed either in collaboration with or by a proxy (QOLP-PD, communication questionnaire [36]; Rosser classification, NHP, measure of goal fulfilment [33]). Six of the studies did not explicitly state who completed the tools used to collect PROMs (QUEST [35]; QUEST and PIADs [40]; author developed tool [27]; MQoL and CETI-M [31]).

## Discussion

This review confirmed that there are no PROMs specifically developed and evaluated for capturing outcomes from people who use AAC. Of the PROMs that have been used with people who use AAC, a range of concepts were measured but there was little or no evidence that any of the tools used had been validated for people who use AAC. PROMs were not consistently completed by the study participants and there was scant explanation about any adaptations that had been made to enable participants to engage directly with the tools. The strength of the findings of the research studies in this review is limited by the often-poor quality of reporting. Nevertheless, some insights about the tools used, the content of these tools and the authorship of PROMs in people who use AAC have been identified.

### Tools

The adaptations to existing, validated PROMs and the use of author-developed tools in studies identified by this review indicate that, despite the lack of suitable tools for use with people who use AAC, attempts are being made to capture patient-reported outcome data. A review by Enderby [20] also found that there was no single clinician-reported outcome measure that was consistently used by AAC services within the UK, but that a range of tools had been adopted and adapted for use with this population. The adaptation of tools in both clinical settings and research studies could indicate that, despite existing tools being insufficient or unavailable, clinicians and researchers are motivated to capture patient-reported outcome data. The current strategic drivers in healthcare in England are focused on empowering patients to become more involved in healthcare decision making [11]. These strategies are likely to lead to an increased demand on health services to capture the impact of greater patient involvement, using tools such as PROMs. Increased interest from clinicians, researchers and policy makers in patient involvement will drive the demand for psychometrically robust PROMs. Greater use of PROMs would increase the involvement of the person who uses AAC in decision making during assessment and also improve the viability of PROMs in the metrics for evaluation.

### Content

One of the critical measures of adequacy of PROMs is the conceptual framework and the description of the relationships between the items (or domains) and concepts being measured [12]. The papers included in this review reported a range of tools that captured a large number of different concepts. The heterogeneity of the PROMs used to capture various aspects of AAC function and use within this review exemplifies one of the challenges of PROMs and AAC—identifying the nature of preferred outcomes. There is a lack of clarity about what outcomes are important to people who use AAC [21] and a range of factors that can affect outcomes. Are positive outcomes in people who use AAC concerned with the function or use of the AAC devices specifically? Or are outcomes concerned with the impact that using AAC has, e.g. on communication, relationships, taking part in activities or independence? Several papers used more than one PROM. One possible advantage of using a range of measures to record PROMs is the opportunity to capture the range of outcomes. This may help researchers to see the “big picture” and illuminate reports about people who use AAC by highlighting ‘that AAC in and of itself is not an end goal… [but] can be used as a tool to achieve other goals’ [21, p. 295]. It is also important to acknowledge that a range of factors can affect outcomes in people who rely on AAC including the patient’s milieu, personality, cognitive skills and the technology of the device itself [38]. A clearer understanding about what constitutes important outcomes from the people who use AAC and in what context, is needed in order to evaluate whether the tools used in research reflect the priorities of the end users.

### Authorship

The poor reporting of authorship (i.e., who completed the PROM) in this review disguises some of the challenges experienced by people who use AAC and therefore may limit validity of the results. Where studies reported that the researcher completed PROM questionnaires, there was no reflection on how this may have influenced the results. In studies where participants had completed PROMs, there was little detail about how this was made possible. It is important to note here that the studies that paid attention to reporting, in detail, the collaborative nature of the PROM authorship were rated as higher quality papers overall on the MMAT tool. Hamm and Mirenda [36], for example, provide details about the extent to which the participants were able to complete the questionnaires independently and discussed the limitations inherent in asking questions of people who have limited functional speech. With a research population that cannot engage easily in traditional methods of data collection, the trustworthiness of the study is strongly correlated with how well adjustments and modifications to methods are reported.

### A note about Talking Mats™

It is of note that there are no papers reporting the use of Talking Mats™ as a patient-reported outcome measure included in this review. Talking Mats™ is a collaboratively produced, picture or text-based tool that has been used to gather opinions and feedback from people who have communication difficulties in both research and service settings [42]. A report using Talking Mats™ was identified by the initial search terms [41] but was excluded from the review during full-text screening process. This was because data relevant to the review population could not be disambiguated. Talking Mats™ has the unusual quality of being used as an AAC device, a research tool and an outcome measure in a range of different studies. It is not a PROM per se as the content is not fixed and therefore not based on a conceptual framework [12], nor has it been psychometrically evaluated with a specific population. It does, however, meet the requirements of a large proportion of people who use AAC in ways that the PROMs reported in this review do not. It is a flexible tool that allows for the use of text or pictures. It is available in paper and digital forms and can be completed by the person who uses AAC, in collaboration with a communication partner or with a trained facilitator who can work with an individual who is employing multiple-modalities to communicate [42]. In the absence of a suitable PROM for people who use AAC, a tool that incorporated some of the flexibility and accessibility of Talking Mats™ could be a useful resource for professionals and services supporting people who use AAC.

### Limitations

The overall quality of the reports identified by this review was relatively low which had an impact on the amount of data that could be extracted. Information about authorship and the conceptual frameworks for PROMs was variably reported, so the discussion of these areas is based on the small amount of data that it was possible to extract. In several of the papers excluded during screening, the research team were unable to disambiguate data about populations or age groups of interest from the reports available. As a result, there was insufficient data available to carry out a sub-group analysis of people 12 to 18 years old as was planned in the original review protocol. The search strategy excluded people who had severe intellectual disability, autism or significant cognitive impairment as there is an additional layer of complexity in using PROMs with these populations which is beyond the scope of this review. These populations do constitute a significant number of people who use AAC however and there would be value in exploring the PROMs employed with these groups in a separate review. The search strategy for this review was large, including terms relating to communication impairments and assistive technology, in order to capture PROMs in populations with similar difficulties that may be of interest or value in the field of AAC. Following title and abstract screening, too many of these reports were identified to be included in this review (see Prisma diagram). The review team decided that this data may instead be useful in a complementary review to be analysed at another point in time.

## Conclusion

This review has identified that there is no single patient-reported outcome measure suitable for use with people who use AAC. No tool was identified by this review that has a conceptual framework specifically for AAC, and of the tools that have been used, there was limited evidence that the necessary adaptations were made to accommodate the multi-modal nature of communication in people who use AAC. Clinical services that provide AAC and support people to use AAC cannot therefore consistently capture outcome data from the patient-perspective. Future investigation into whether or not the domains generated during this review, from the tools that have been used with people who use AAC, reflect outcomes that are important to this population will be necessary.

PROMs can be valuable tools to aid understanding of the impact of a condition, treatment or intervention from the patient’s perspective. They can also improve patient-provider communication by facilitating discussion, supporting decision-making and clarifying shared aims for treatment. When selecting a PROM, the conceptual frameworks of the tool need to adequately reflect the priorities of the population of interest. There is currently no consensus about the important outcomes of AAC from the perspective of the people who use it. The population of people who use AAC have a particular set of needs and requirements to enable their inclusion in clinical decision making in healthcare and in research. The multi-modal and collaborative nature of communication by and with people who use AAC should be accommodated by developing PROMs that adequately reflect the needs and priorities of this population. Acknowledging the adaptations that have been made during research studies to accommodate people with additional needs, such as accurately describing authorship in people with communication difficulties, is essential if the results are to be considered authentic and trustworthy.
